# The placenta as a cradle, but not source, of blood?

**DOI:** 10.1371/journal.pbio.3003021

**Published:** 2025-02-06

**Authors:** Julie Y. Chen, Kyle M. Loh

**Affiliations:** Department of Developmental Biology, Institute for Stem Cell Biology and Regenerative Medicine, Stanford University, Stanford, California, United States of America

## Abstract

An important question is whether the placenta is a source of, or merely a niche for, blood-forming hematopoietic stem cells. This Primer highlights a recent PLOS Biology study showing that the placenta does not directly give rise to hematopoietic stem cells.

## Teaser

An important question is whether the placenta is a source of, or merely a niche for, blood-forming hematopoietic stem cells. A recent *PLOS Biology* study suggests that the placenta does not directly give rise to hematopoietic stem cells.

Every day, the body produces billions of new blood and immune cells to replace those lost to daily attrition. Manufacturing of new blood and immune cells occurs at a vast scale and an unprecedented pace, and is fueled by blood-forming hematopoietic stem cells (HSCs) [1]. As such, a timeless question in developmental biology concerns how and whence HSCs arise in the embryo [2]. Namely, how is the foundation of the future blood and immune system laid down during early development?

Within the embryo’s blood vessels, arterial endothelial cells give rise to HSCs [3], but an important issue remains unresolved. Do all arteries form HSCs? Or are only some arteries ordained with the responsibility of producing HSCs? The first major artery within the embryo, known as the dorsal aorta, likely produces HSCs [2]. However, can other arteries similarly give rise to blood?

The quest to define the developmental origins of blood has recently turned to the placenta. Ensconced deep within the mother’s womb, human and mouse embryos cannot breathe, and they thus construct supporting structures—the umbilical cord and placenta—to interface with the mother to acquire oxygen and nutrients, and to discharge carbon dioxide [4]. The embryo is connected via the umbilical cord to the placenta, which in turn is physically adhered to the mother’s uterus. This intimately juxtaposes the respective blood vessels of the embryo and the mother, thereby enabling lifesaving gas and nutrient exchange to occur [4].

Interestingly, the placenta physically harbors HSCs [5,6]. This observation piqued the curiosity of many. Does the placenta merely act as a landing pad for itinerant HSCs that arose from other embryonic locations and traveled through the circulation to take up residence in the placenta? Or alternatively, do HSCs emerge directly from the placenta? The placenta is densely infiltrated by arteries that effectuate gas exchange between the embryo and the mother [4], and arteries are known to form HSCs [3]. Indeed, in 2008, Rhodes and colleagues proposed that the placenta might directly generate HSCs [7].

**Fig 1:**
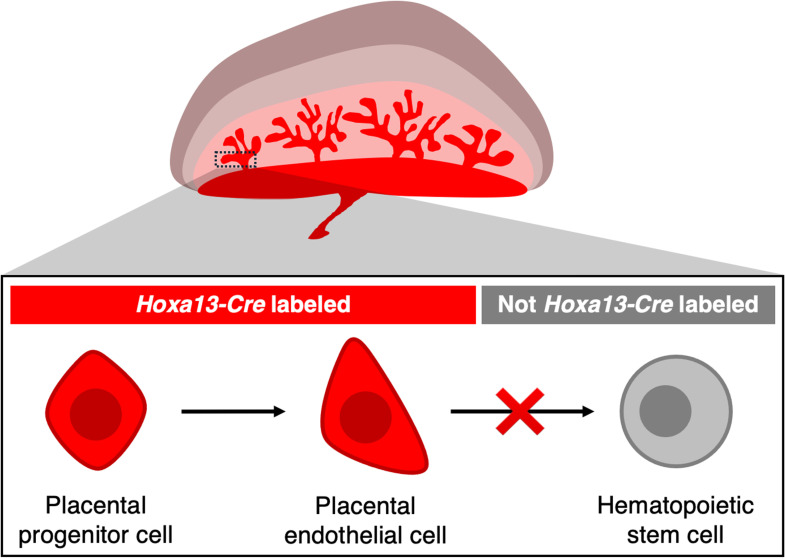
The placenta is unlikely to generate blood-forming hematopoietic stem cells (HSCs). In mouse embryos, *Hoxa13*-*Cre* lineage tracing labels virtually all endothelial cells within the mouse placenta. However, virtually no HSCs are labeled by *Hoxa13-Cre*. This suggests that the placenta is unlikely to form HSCs. Rather, these results suggest that HSCs are produced elsewhere in the developing embryo and subsequently migrate to the placenta, which serves as a landing pad for HSCs.

In a recent *PLOS Biology* study, Chen and Tober and colleagues employ genetic lineage tracing to rigorously address the longstanding question of whether the placenta forms HSCs [8] (Fig 1). The precursor cells of placental vasculature express the *Hoxa13* gene [9]. The authors thus employ a *Hoxa13-Cre* genetic lineage tracing system to permanently label placental vasculature and all their progeny cells. This *Hoxa13-Cre* system allowed the authors to test if placental vasculature forms HSCs: when cells within the mouse embryo express *Hoxa13*, they also express Cre recombinase, which permanently labels cells with a fluorescent protein marker [9]. This genetic lineage tracing approach is elegant because placental endothelial cells and all their future progeny cells are permanently labeled: even if cells divide, migrate, or even turn off *Hoxa13* expression, they will retain the fluorescent protein marker. Another strength of genetic lineage tracing is that it is non-invasive: living cells within the mouse embryo are genetically labeled, without the need to dissociate or culture the tissue, transplant cells, or physically inject cells with a dye.

The *Hoxa13-Cre* system labels essentially all placental endothelial cells with the fluorescent protein [9]; in striking contrast, virtually no HSCs within the placenta or other parts of the embryo (namely, the fetal liver or bone marrow) express the fluorescent protein [8] (Fig 1). Placental vasculature is thus unlikely to be a major source of HSCs. Instead, HSCs may predominately arise from alternative cellular sources, perhaps other arteries, including the dorsal aorta.

Interestingly, a minute number of HSCs might be labeled by *Hoxa13-Cre*, which may reflect HSCs arising from umbilical cord endothelial cells [8]. Because *Hoxa13-Cre* labels both placenta and umbilical cord vasculature [9], it is still unclear whether the putative *Hoxa13-Cre*-labeled HSCs emanate from the placenta or umbilical cord. Future work may require placenta- versus umbilical cord-specific markers to distinguish the two possibilities.

Additionally, the authors show that *Runx1*, a marker gene of endothelial cells transitioning into blood cells, is not expressed in endothelial cells from mouse or human placentas. This suggests that placental vasculature does not form blood cells in either mouse or human embryos.

How can the present study be reconciled with earlier work that suggested a placental origin of HSCs [7]? The present study uses genetic lineage tracing to label placental endothelial cells within their native tissue and to test if they form HSCs in vivo. Meanwhile, a previous study [7] focused on the different, but related, question of what placental cells can do when placed in the admittedly artificial environment of cell culture. In the previous study [7], placentas from *Ncx1*-knockout mouse embryos—which apparently lack a circulation—were studied to ask if blood and immune cells are directly produced by the placenta, as opposed to arising from other locations and traversing the circulation to enter the placenta. The placenta was separated from either wild-type or *Ncx1*-knockout mouse embryos, dissociated into single cells, and then cultured, whereupon blood and immune cells arose in vitro [7]. However, this approach did not formally demonstrate that the placenta formed HSCs, which would require stringent proof that a single cell—namely, an HSC—produces multiple types of blood and immune cells in vivo [1]. Additionally, it is possible that prior to cell culture, the process of physically separating the placenta from the mouse embryo inadvertently introduced cells from other embryonic tissues, which generated the blood and immune cells observed in the culture.

Overall, the present authors’ data support a model wherein HSCs are generated elsewhere within the embryo, and then migrate to the placenta, which may serve as a niche for HSCs. Why should this be the case? Following Theodosius Dobzhansky’s maxim that “nothing in biology makes sense except in the light of evolution,” the authors supply an interesting evolutionary observation [8]. HSCs arose in animal evolution prior to the placenta [10]. Perhaps, HSCs were first produced from an ancestral source (e.g., the dorsal aorta) in early animals, and subsequently, once the placenta was created in later animals, HSCs “learned” to migrate to the placenta and to take up temporary residence there [8]. If so, maybe the placenta serves as the cradle for, but not the fount of, blood.
